# Modified fenestrated/branched endovascular aortic repair with short bridging stent to treat complex aortic dissection

**DOI:** 10.3389/fcvm.2024.1496139

**Published:** 2024-11-07

**Authors:** Zihe Zhao, Yuexue Han, Reyaguli Keyoumu, Shuai Zhang, Xia Gao, Zhao Liu

**Affiliations:** ^1^ Department of Vascular Surgery, Nanjing Drum Tower Hospital, Affiliated Hospital of Medical School, Nanjing University, Nanjing, China; ^2^ Jiangsu Provincial Key Medical Discipline (Laboratory), Department of Otolaryngology Head and Neck Surgery, Nanjing Drum Tower Hospital, Affiliated Hospital of Medical School, Nanjing University, Nanjing, China

**Keywords:** aortic dissection, fenestrated/branched endovascular aortic repair, physician-modified stent graft, short bridging stent, 3D-printing

## Abstract

**Objectives:**

This study aims to improve fenestrated/branched endovascular aortic repair (F/B EVAR) through fabricating physician-modified stent grafts (PMSG) with short bridging stent to treat complex aortic dissection.

**Methods:**

From November 2018 to January 2024, a total of 82 aortic dissection patients were treated by F/B EVAR combined with short bridging stents, including 19 aortic arch dissection patients and 63 thoracoabdominal aortic dissection patients. Inner or outer short bridging stents were applied to fabricate PMSG with the help of 3D-printing models intraoperatively. All patients underwent postoperative evaluation by enhanced computed tomography in follow-up.

**Results:**

All aortic dissections were successfully repaired. In aortic arch group, the average operative time was 289.2 ± 88.8 min. The perioperative mortality rate was 5.3%. The total reintervention rate was 5.3%. The average follow-up duration of 36.2 ± 9.5 months. The total incidence of endoleak after surgery was 15.8%. In thoracoabdominal aorta group, the average operative time was 345.5 ± 112.0 min. The perioperative mortality rate was 1.6%. The total reintervention rate was 1.6%. The average follow-up duration of 32.4 ± 19.2 months. The total incidence of endoleak after surgery was 11.1%.

**Discussion:**

The application of short bridging stents has shown promising results in reducing endoleak rates after F/B EVAR. 3D-printing is a feasible way to assist the precise fenestration and design of short bridging stents. However, the safety and reliability of this method need to be further validated.

## 1 Introduction

Aortic dissection (AD) is one of the most common life-threatening aortic diseases that often requires timely surgical intervention (1, 2). The complex aortic dissection, which involved major arterial branches, usually creates intricate anatomical challenges for surgeons. Currently, the main treatments for aortic dissection include open surgery and endovascular aortic repair (EVAR) (3). In contrast with open surgery, EVAR shows shorter hospital stays, lower complication rates, and reduced mortality (4). With the advancement of complex endovascular techniques, fenestrated/branched endovascular aortic repair (F/B EVAR) has been developed to address complex aortic dissection involving branch arteries (5). F/B EVAR can be applied in complex anatomical conditions, restoring blood flow in a manner consistent with normal physiology (6, 7). However, F/B EVAR also faces challenges, such as high incidences of type I and III endoleaks particularly in multiple fenestrations. Moreover, F/B EVAR is primarily used for the repair of aortic aneurysms (8, 9), with less practice and reporting in the repair of AD.

This study improved the traditional F/B-EVAR by combining short bridging stents with the main body stent graft to create physician-modified stent grafts (PMSG) with the help of 3D printed model. For complex aortic dissections involving multiple branch arteries, the PMSGs with short bridging stents has shown to effectively repair dissection and reduce the incidence of endoleaks after F/B-EVAR.

## 2 Materials and methods

### 2.1 General information of patients

From November 2018 to January 2024, a total of 82 AD patients were treated by F/B EVAR combined with short bridging stent. The inclusion criteria for patients were as follows: (1) Dissection involving the branches of the arch or abdominal aorta; (2) The entry tear of the dissection was less than 1 cm away from the involved branch artery; (3) Patients have no contraindications to F/B EVAR; (4) Patients did not undergo EVAR before. The exclusion criteria included: (1) The entry tear of the dissection was 1 cm or more away from the involved branch artery; (2) Patients who were pregnant; (3) Patients with other serious diseases (such as tumors or severe infections); (4) Patients who refused F/B EVAR. Finally, 19 patients (17 males and 2 females) with aortic arch dissections involving branches, were included in aortic arch group. 63 patients (58 males and 5 females) with thoracoabdominal aortic dissections involving the visceral branches, were included in thoracoabdominal aorta group.

The baseline characteristics of patients in aortic arch group and thoracoabdominal aorta group were depicted in Table 1 and Table 2. Surgical duration, intraoperative blood loss, contrast agent volume, post-operative intensive care duration, and hospital stay of all patients were recorded. Perioperative and follow-up mortality, the incidence of complications like endoleaks, and the rate of re interventions were retrospectively analyzed. This study was approved by the ethics committee of Nanjing Drum Tower Hospital, affiliated with Nanjing University Medical School. Informed consent was obtained from all participants.

**Table 1:** Baseline characteristics of aortic arch patients.

Variable	No. (%) or mean ± standard deviation (*N* = 19)
Gender (%)	
Male	17 (89.5)
Female	2 (10.5)
Age (year)	56.9 ± 13.1
Hypertension (%)	15 (78.9)
Diabetes (%)	3 (15.8)
Coronary artery disease (%)	4 (21.1)
Hyperlipoidemia (%)	9 (47.4)
Renal insufficiency (%)	1 (5.3)
Smoking (%)	16 (84.2)
Average follow-up (months)	36.2 ± 9.5

Data presented as mean ± standard deviation for continuous variables and number (%) for categorical variables.

**Table 2:** Baseline characteristics of thoracoabdominal aortic patients.

Variable	No. (%) or mean ± standard deviation (*N* = 63)
Gender (%)	
Male	58 (92.1)
Female	5 (7.9)
Age (year)	52.5 ± 9.9
Hypertension (%)	56 (88.9)
Diabetes (%)	20 (31.7)
Coronary artery disease (%)	8 (12.7)
Hyperlipoidemia (%)	43 (68.3)
Renal insufficiency (%)	4 (6.3)
Smoking (%)	48 (76.2)
Average follow-up (months)	32.4 ± 19.2

Data presented as mean ± standard deviation for continuous variables and number (%) for categorical variables.

### 2.2 Preparation of 3D printed models

**Figure 1:**
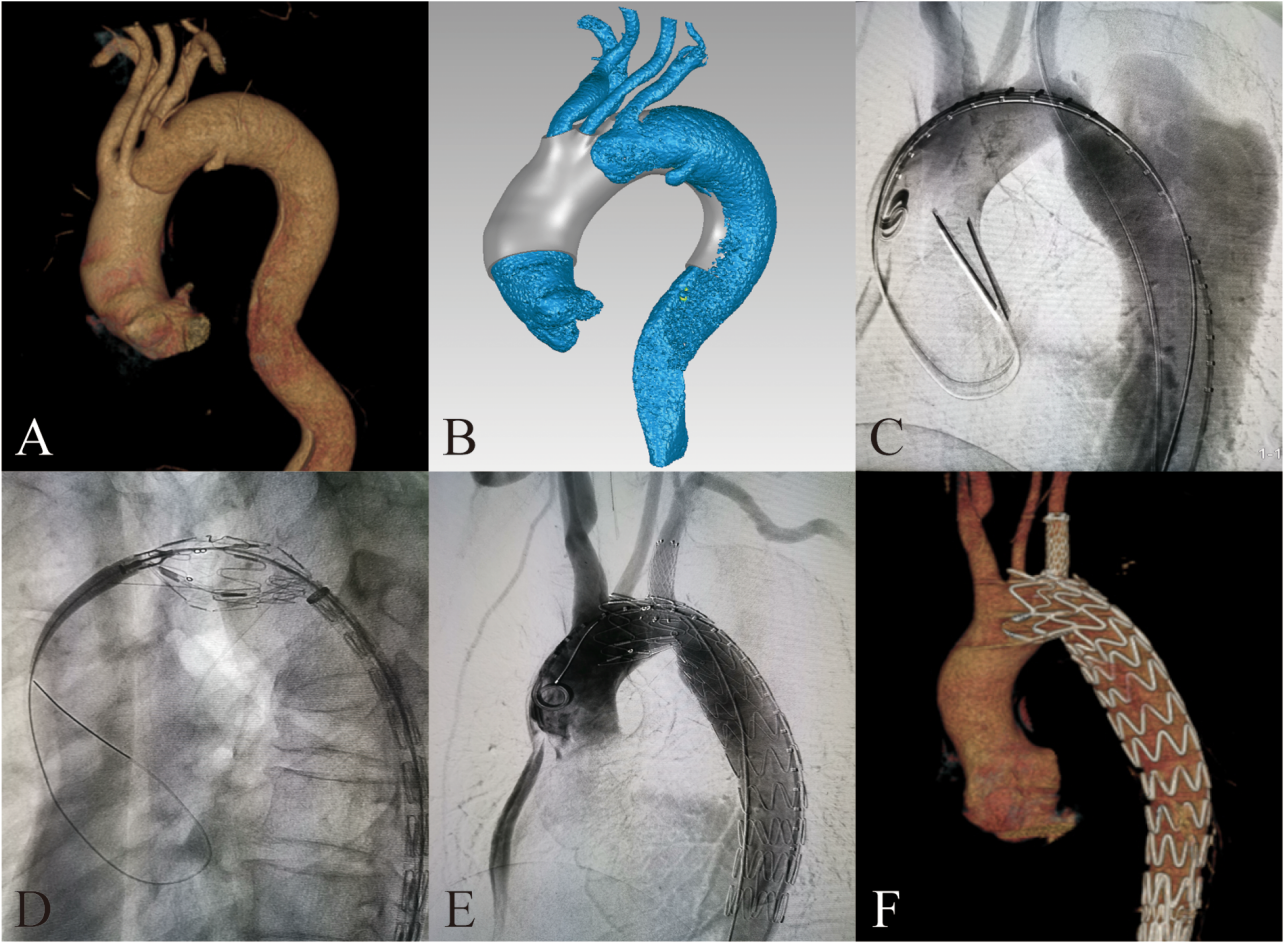
3D-printing assists in fabrication of PMSG with short bridging stents to repair aortic arch dissection. **(A)** Preoperative 3D reconstruction of the aortic arch dissection based on CTA. **(B)** Design of 3D-printing model. **(C)** Preoperative DSA imaging of the aortic arch dissection. **(D)** Delivery sheath enter into the fenestration. **(E)** Postoperative DSA imaging of the aortic arch dissection. **(F)** 3D reconstruction of the aortic arch dissection at 3 months after surgery.

**Figure 2:**
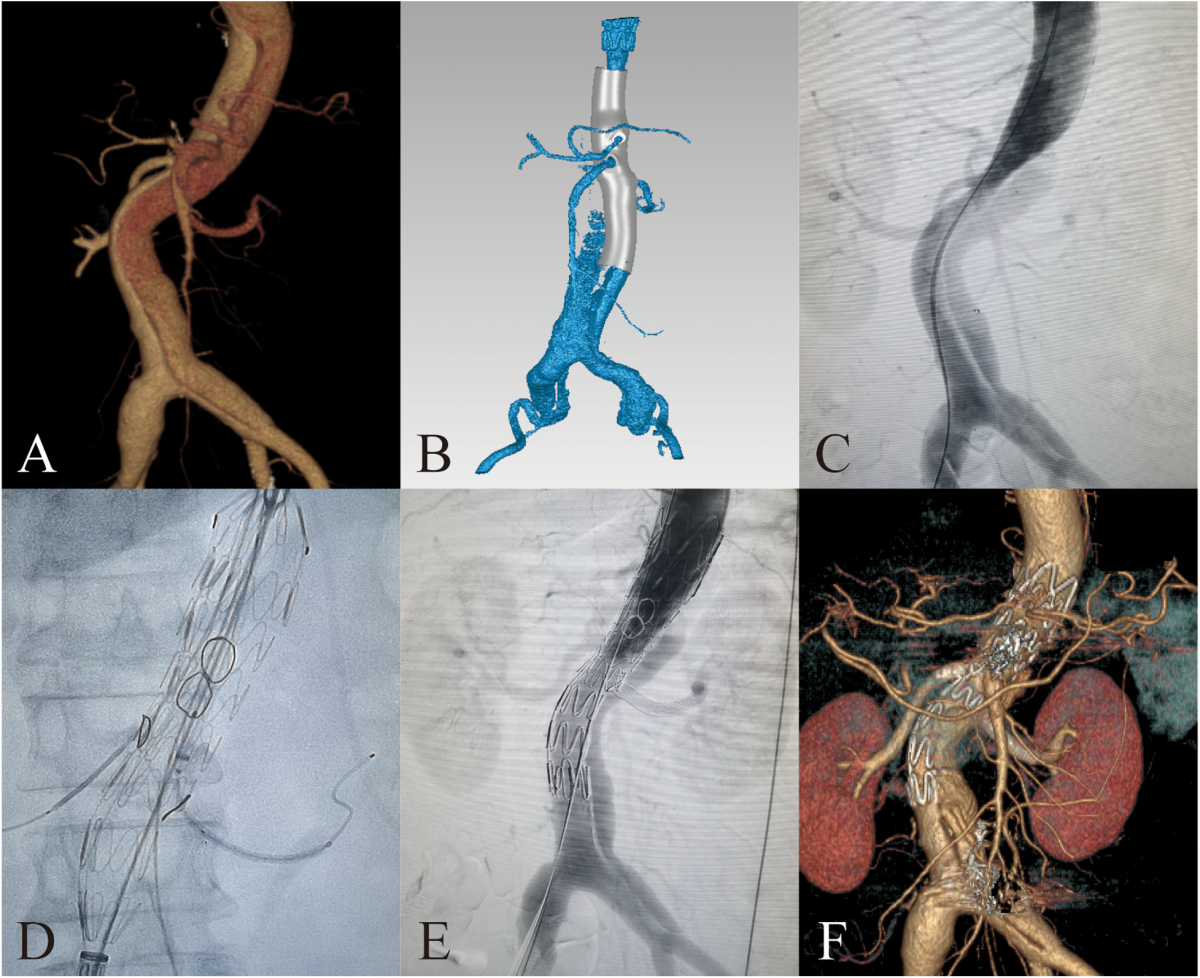
3D-printing assists in fabrication of PMSG with short bridging stents to repair thoracoabdominal aortic dissection. **(A)** Preoperative 3D reconstruction of the thoracoabdominal aortic dissection based on CTA. **(B)** Design of 3D-printing model. **(C)** Preoperative DSA imaging of the thoracoabdominal aortic dissection. **(D)** Delivery sheath enter into the fenestration. **(E)** Postoperative DSA imaging of the thoracoabdominal aortic dissection. **(F)** 3D reconstruction of the thoracoabdominal aortic dissection at 3 months after surgery.

All patients underwent aortic computed tomography angiography (CTA) to obtain original radiological data (Figures 1A, 2A, slice thickness 0.5 mm). 3D reconstructions of the aorta were performed by Mimics software version 21.0 (Materialise, Belgium). Subsequently, we conducted reverse simulation analysis of vascular deformation using Geomagic Design software (3D Systems, USA) to create digital 3D models. Combined with surgical plans, preprocedural fenestrations were designed and determined at the main body stent graft (Figures 1B, 2B). The model data were then exported to a 3D printer (Eden260VS, from Stratasys, USA) to fabricate the 3D printed models using biocompatibility material, which were sterilized with ethylene oxide.

### 2.3 Intraoperative modification of PMSGs

We selected appropriate aortic covered stents (from Lifetech or Medtronic) and branch artery stents (Viabahn by W.L. Gore & Associates, Fluency by Bard Peripheral Vascular, Omnilink by Abbott and SilverFlow by Lifetech) based on the preoperative measurements. For aortic dissections, the oversizing of main body stent grafts should be 5% to 10% of the diameter of the aorta at the anchoring zone. The aortic main body stent graft is fully released inside the transparent 3D-printed model. Fenestration markings are made by marker pens and placed between metal edges to minimize the impact of expansion after branch stent implantation.

**Figure 3:**
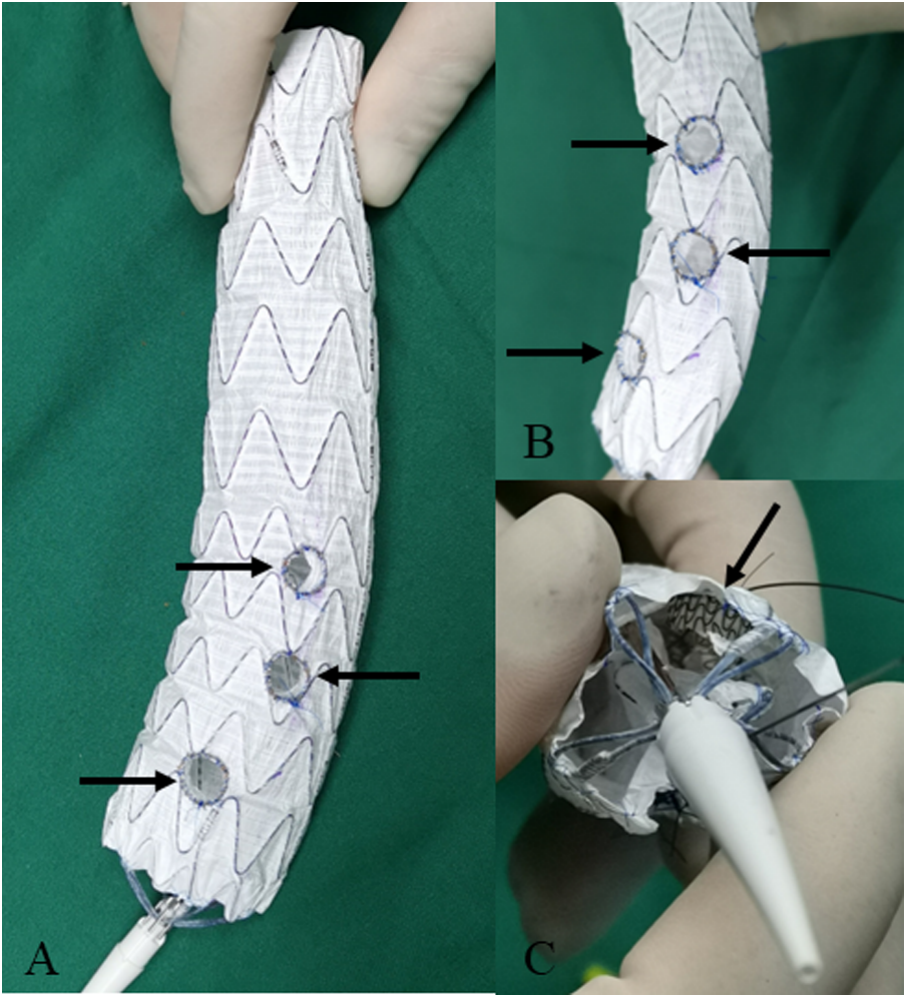
Modifying the fenestrated main body stent graft with short bridging stents for the repair of aortic arch dissection. **(A,B)** The inner short bridging stents (indicated by arrow) are sutured at the fenestration. **(C)** The inner short bridging stents are indicated by arrow (inside view).

**Figure 4:**
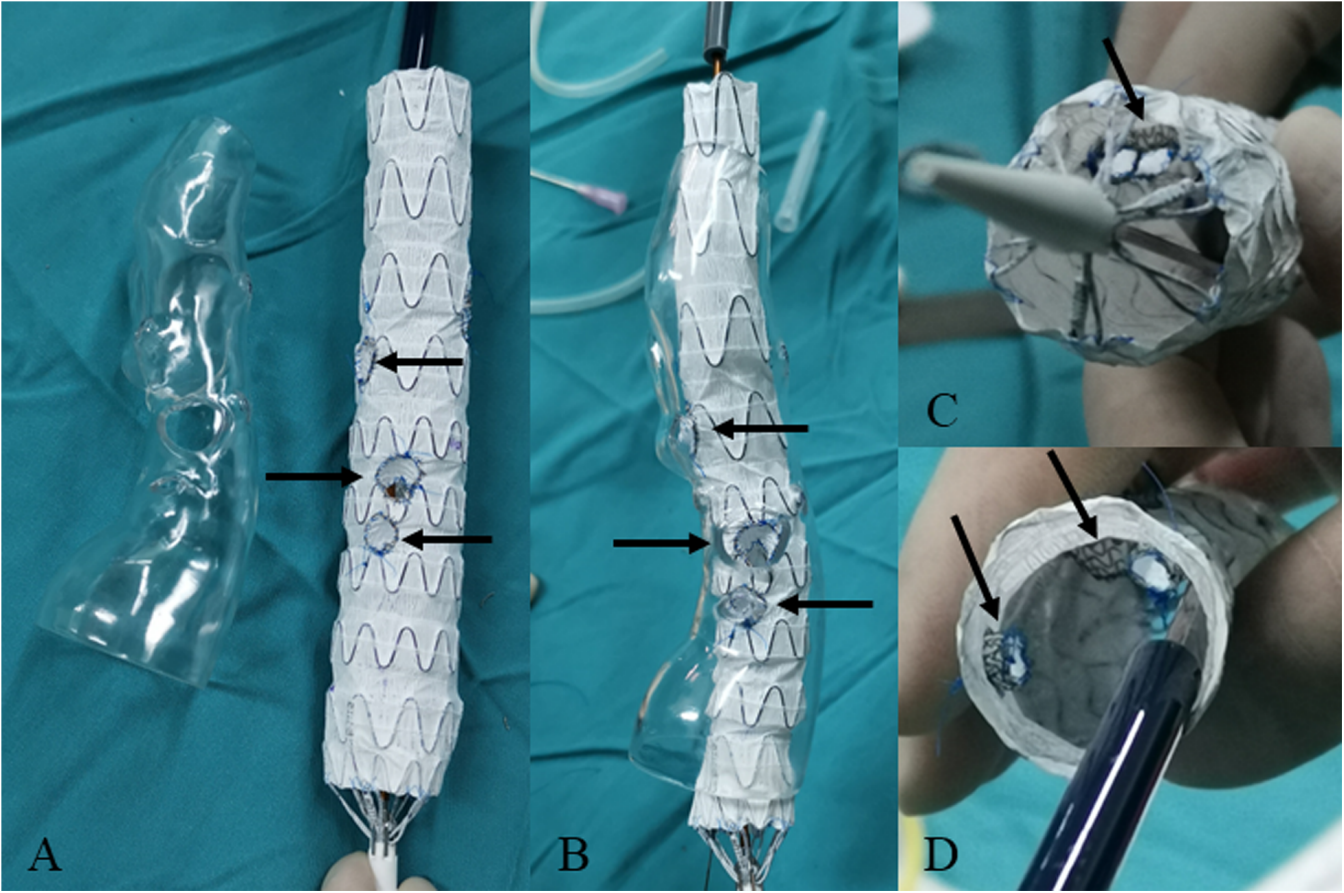
Modifying the fenestrated main body stent graft with short bridging stents for the repair of thoracoabdominal aortic dissection. **(A)** The fenestration (indicated by arrow) of main body stent graft based on the 3D-printed model. **(B)** Releasing the fenestrated main body stent graft in the lucid 3D-printed model. The outer short bridging stents are indicated by arrow. **(C,D)** The inner short bridging stents are indicated by arrow (inside view).

For dissection at aortic arch, we employed inner short bridging stents as short bridging stents due to the approach from the branch artery to the aorta. We trimmed the covered stents (Viabahn, Gore, USA) to a length of 3–5 mm, with the inner branch diameter being 1–2 mm smaller than the implanted branch stents. The inner short bridging stent was placed inside the fenestrated main body stent graft. Spring coils was sutured using vascular sutures as selection markers at the fenestrations (Figure 3). For dissection at thoracoabdominal aorta, we employed inner or outer short bridging stents as short bridging stents according to the anatomical condition. For anatomically straighter branches, we chose short outer bridging stents. We sutured the covered stents (Viabahn, Gore, USA) with the fenestrated main body stent graft at the external window using spring coils and vascular sutures. Then we trimmed the outer short bridging stents to 3–5 mm. For aortic lumen narrowing with twisting, we employed inner short bridging stents as short bridging stents. We trimmed the covered stents (Viabahn, Gore, USA) to a length of 10–15 mm, with the inner branch diameter being 1–2 mm smaller than the implanted branch stents. Then, we sutured spring coils at the internal entrance of inner short stents as intraoperative selection markers. Finally, we used non-absorbable sutures to reinforced the inner short bridging stent to prevent intraoperative displacement of the short bridging stent. To facilitate the selection of fenestration, we used vascular sutures and a 0.018-inch guidewire on one side of the main body stent graft to reduce the diameter of it by approximately 20%–30% (Figure 4).

### 2.4 Surgical procedure

For patients with dissection at aortic arch, access was obtained through the femoral artery, left brachial artery, left common carotid artery, and right axillary artery (or right brachial artery). We delivered the PMSG to the preoperatively planned position in the aortic arch via the femoral artery approach. Then we slowly released the anterior segment of the main body stent graft. Through each branch artery approach, with the stent in its constricted state, catheters were inserted and selectively advanced into their respective fenestrations sequentially from the brachiocephalic artery, left common carotid artery, to the left subclavian artery. After inserting a long delivery sheath, we pulled out the constraining wire and fully released the main body stent graft. Then respective branch stents were placed according to different diameters of branch arteries. We dilated expansion balloons to prevent endoleak at the bridging sites. Finally, ascending aorta and aortic arch angiography are conducted to confirm the patency of each branch artery and to check for endoleaks (Figures 1C–F).

For patients with thoracoabdominal aortic dissection, access was typically obtained through the right femoral artery, left axillary artery, and left femoral artery. Based on preoperative assessment, one femoral artery served as the access for the PMSG. A 16–18F long delivery sheath (Gore Dryseal Flex) was inserted into the left axillary artery as the access for the visceral artery branch stent. In a partially released state of PMSG, each branch artery is sequentially selected through the preprocedural fenestrations. The guide wire and catheter were placed outside the external opening through the internal opening before selecting the visceral branch arteries. Then branch artery stents were implanted along the wire and released. Bridging sites were routinely dilated with an expansion balloon. Finally, additional aortic stents were implanted at the distal or proximal end of the fenestrated stent to completely repair the thoracoabdominal aortic dissection (Figures 2C–F).

## 3 Results

A total of 19 patients underwent aortic arch F/B EVAR using PMSGs. The average operative time was 289.2 ± 88.8 min. A total of 50 target branch arteries were reconstructed. Among these, 1, 5 and 13 patients respectively underwent single fenestration, double fenestrations and triple fenestrations. A total of 49 branch artery stents were implanted, including 27 Viabahn (W.L. Gore & Associates) and 22 Fluency (Bard Peripheral Vascular) stents. 48 short bridging stents were sutured at the fenestration sites, all of which were inner short bridging stents using Viabahn (W.L. Gore & Associates) stents.

In aortic arch group, the outcome measures were listed in Table 3. Intraoperative blood loss was 100 (IQR = 350) ml. Contrast medium use was 183.6 ± 47.3 ml. Postoperative ICU stay was 0.9 ± 1.4 days. Postoperative hospital stay was 7.7 ± 3.6 days. Postoperative contrast-enhanced CT scans showed patency in all branch arteries above the aortic arch. The perioperative mortality rate was 5.3%. The total reintervention rate was 5.3%. One patient suffered sudden postoperative death of unknown cause. Another patient developed retrograde dissection postoperatively and underwent open surgery. All patients in aortic arch group were followed up and had an average follow-up duration of 36.2 ± 9.5 months. During follow-up, no patient deaths occurred. One patient experienced retrograde dissection and was treated with open surgery. The incidence of endoleak during the perioperative period and follow-up was 15.8%. Two patients suffered endoleaks during the perioperative period, including one Type Ia and one Type Ic. During postoperative follow-up, one patient had a Type II endoleak from the bronchial artery, which was cured after treatment with coil embolization. No Type I or Type III endoleaks were observed during follow-up. No other serious complications occurred.

**Table 3:** Outcome measures of the modified F/B EVAR.

Outcome measures	Aortic arch group (*N* = 19)	Thoracoabdominal aorta group (*N* = 63)
Intraoperative blood loss (ml)	100 (350)	300 (450)
Contrast medium use (ml)	183.6 ± 47.3	185.2 ± 44.5
Postoperative ICU stay (days)	0.9 ± 1.4	1.0 ± 0.8
Postoperative hospital stay (days)	7.7 ± 3.6	8.4 ± 4.6
Perioperative mortality rate (%)	5.3	1.6
Total reintervention rate (%)	5.3	1.6
Total incidence of endoleak (%)	15.8	11.1
Average follow-up duration (months)	36.2 ± 9.5	32.4 ± 19.2

Data presented as mean ± standard deviation or median (IQR) for continuous variables.

A total of 63 patients in thoracoabdominal aorta group underwent F/B EVAR using PMSGs. Among them, 2, 21 and 40 patients respectively underwent double fenestration, triple fenestration and quadruple fenestration. The average operative time was 345.5 ± 112.0 min, with no cases converting to open surgery. A total of 227 branch arteries were reconstructed. A total of 225 branch stents were implanted, including 163 Viabahn (W.L. Gore & Associates), 26 Fluency (Bard Peripheral Vascular), 28 Omnilink (Abbot), and 8 SilverFlow (Lifetech). 174 short bridging stents were sutured at fenestrations, including 16 inner short bridging stents and 158 outer short bridging stents. All short bridging stents were Viabahn (W.L. Gore & Associates).

In thoracoabdominal aorta group, the outcome measures were listed in Table 3. Intraoperative blood loss was 300 (IQR = 450) ml. Contrast agent use was 185.2 ± 44.5 ml. Postoperative ICU stay was 1.0 ± 0.8 days. Postoperative hospital stay was 8.4 ± 4.6 days. Postoperative contrast-enhanced CT scans showed patency in all visceral branch arteries, with a significant increase in true lumen diameter and a decrease or disappearance of the false lumen compared to preoperative measurements. The perioperative mortality rate was 1.6%. The reintervention rate was 1.6%. One patient died due to liver failure 3 days postoperatively. One patient developed paraplegia, which cured after conservative treatment. One patient experienced acute renal failure postoperatively, considered to be drug-induced. All patients were followed up. The average follow-up duration was 32.4 ± 19.2 months. No postoperative complications related to spinal cord ischemia, intestinal ischemia, or renal and other visceral artery ischemia were observed. One patient developed a subcapsular renal hematoma during follow-up, which improved after conservative treatment. One patient experienced renal artery occlusion one year postoperatively. One patient suffered acute renal failure caused by acute thrombosis of both renal arteries one year postoperatively and cured after emergency surgery. No other serious complications occurred. The incidence of endoleak during the perioperative period and follow-up was 11.1%. During the perioperative period, one case of Type IIIb endoleak occurred due to the proximal barbs of the main body stent graft puncturing the fenestrated main body stent graft, which was cured after coil embolization. Endoleaks in six patients were observed during follow-up. Two cases suffered Type Ib endoleak from the distal tear of the aortic dissection, which were repaired with reintervention on the distal abdominal aorta and iliac arteries. One case was Type Ic endoleak. Two cases were Type IV endoleak. One patient experienced a Type IIIc endoleak due to branch stent dislodgment two years postoperatively, which was repaired after endovascular reintervention.

## 4 Discussion

F/B EVAR allows for the reconstruction of branch arteries according to the specific anatomical features of the aortic dissection, effectively maintaining the patency of these branches. Compared to open surgery, F/B EVAR is associated with lower perioperative morbidity and mortality rates (10–12). Although open surgery was an option especially in the aortic arch group, endovascular repair was more suitable for the patients because the ascending aortae were not involved. However, traditional F/B EVAR is more likely to occur endoleaks at the fenestration bridging sites, leading to reintervention (13, 14). Recently, PMSG has been widely applied in F/B EVAR and achieves favorable outcomes (12, 15–18). However, there are more challenges on stent modification and placement in aortic dissections due to the smaller luminal space compared with aortic aneurysms. Moreover, repair of aortic dissections using PMSGs may lead to endoleaks due to misalignment. Therefore, accurate fenestration alignment to reduce the occurrence of endoleaks has become a crucial issue.

To solve the existing shortages of PMSGs, we improved F/B EVAR. We sutured inner/outer short bridging stents on the main body stent graft, effectively preventing endoleaks at the fenestration. This approach is suitable for dissections due to little space requirement. Given the access from branch arteries above the arch, branch stents can be delivered from the branch arteries to the aorta. We adopted inner short bridging stents in all aortic arch dissection patients. For thoracoabdominal aortic dissections, we adopted inner and outer short bridging stents because branch arteries were selected from within the PMSG outward. If the true lumen was slightly large, we utilized the outer short bridging stent, which were not only simple to fabricate but also capable of sealing the false lumen. If the true lumen was severely compressed or twisted, we utilized inner short bridging stents. Inner short bridging stents did not occupy external space and were easy for selection and alignment. In addition, the inner short bridging stent could support the delivery sheath of branch stents, facilitating entry into branch arteries. To reduce the effect of modification on physical property of PMSGs, we made fenestrations on the covered membrane and avoided the metallic framework. Besides, we reinforced the bridging stents using spring coils. Up to now, no fracture of bridging stents has been observed during the follow-up. The durability of the PMSGs needs to be assessed in a longer follow-up period.

The technique of short bridging stents requires precise preoperative planning and intraoperative fenestration. To overcome the challenges of precise positioning of short bridging stents, we have utilized 3D-printed models to assist in modifying PMSGs. Previously, we have applied 3D-printing technology in the fabrication of PMSGs with favorable outcomes (19, 20). The approach can significantly reduce alignment time and speed up the release procedure of PMSG. Moreover, various commercial aortic stent grafts can be applied to fabricate PMSGs incorporating short bridging stents with the help of 3D printing. The modified procedure required additional materials and extended operative time, likely bringing extra costs. However, F/B EVAR commonly cost less compared to open surgery due to the less surgery cost and lower complication rate. According to our experience, the total cost of F/B EVAR is approximately one-third of that of open surgery in our center.

Endoleak is one of the most common complications following EVAR and significantly impacts prognosis of patients. It is reported that the incidence is approximately 8.2% for Type Ia endoleaks (21), 0 to 8% for Type Ib (22), and around 3.7% for Type III endoleaks (23) after EVAR. Compared to EVAR, F/B EVAR is more likely to occur endoleaks. One study reported that 37.3% of patients experienced endoleaks after F/B EVAR (24). Type I and Type III endoleaks, particularly those occurring at the stent-graft fenestration bridging sites, are more common after F/B EVAR and are challenging to manage (25). About 14% (14.1% (26) and 13.6% (27)) of patients developed Type Ic and Type IIIc endoleaks after F/B EVAR. In our study, the total endoleak incidence was 12.2% after undergoing F/B EVAR with the short branch technique. The incidence of endoleak in aortic arch group is 15.8% and that in thoracoabdominal aorta group is 11.1%. Among these, the incidence is 6.1% for Type I endoleaks and 2.4% for Type III. The combined incidence of Type I and Type III endoleaks is 8.5%. Compared to traditional F/B EVAR, modified F/B EVAR with short bridging stents demonstrates a lower incidence of postoperative endoleaks.

The modified F/B EVAR with short bridging stents has several limitations. Firstly, short bridging stents require surgeons to suture intraoperatively, which extends anesthesia and surgery duration. Second, the trimming and suturing of short bridging stents demand surgical experience, which depends on the skill of surgeons. Thirdly, due to the modification of the covered stent grafts, the durability of the PMSGs and late complications needs to be observed and assessed in a longer follow-up period. Lastly, the generalizability of the results was restricted due to the sample size of the study. The safety and reliability of this method need to be validated in multicenter, large-sample cohorts.

## 5 Conclusion

We have modified traditional F/B EVAR by integrating short bridging stents with 3D-priting technology. By fabricating PMSGs with short bridging stents, we transform line-to-surface contact between stents to surface-to-surface contact, offering an effective approach to reduce endoleaks after F/B EVAR.

## Data Availability

The original contributions presented in the study are included in the article/Supplementary Material, further inquiries can be directed to the corresponding author/s.

## References

[1] Bossone E Eagle KA. Epidemiology and management of aortic disease: aortic aneurysms and acute aortic syndromes. Nat Rev Cardiol. (2021) 18(5):331–48. 10.1038/s41569-020-00472-6 33353985

[2] Erbel R Aboyans V Boileau C Bossone E Bartolomeo RD Eggebrecht H 2014 ESC guidelines on the diagnosis and treatment of aortic diseases: document covering acute and chronic aortic diseases of the thoracic and abdominal aorta of the adult. The task force for the diagnosis and treatment of aortic diseases of the European Society of Cardiology (ESC). Eur Heart J. (2014) 35(41):2873–926. 10.1093/eurheartj/ehu281 25173340

[3] Baman JR Malaisrie SC. What is aortic dissection? JAMA. (2023) 330(2):198. 10.1001/jama.2023.5592 37171803

[4] Liu D Luo H Lin S Zhao L Qiao C. Comparison of the efficacy and safety of thoracic endovascular aortic repair with open surgical repair and optimal medical therapy for acute type B aortic dissection: a systematic review and meta-analysis. Int J Surg. (2020) 83:53–61. 10.1016/j.ijsu.2020.08.051 32927144

[5] Xodo A D'Oria M Mendes B Bertoglio L Mani K Gargiulo M Peri-operative management of patients undergoing fenestrated-branched endovascular repair for juxtarenal, pararenal and thoracoabdominal aortic aneurysms: preventing, recognizing and treating complications to improve clinical outcomes. J Pers Med. (2022) 12(7). 10.3390/jpm12071018 PMC9317732 35887518

[6] Motta F Crowner JR Kalbaugh CA Marston WA Pascarella L McGinigle KL Outcomes and complications after fenestrated-branched endovascular aortic repair. J Vasc Surg. (2019) 70(1):15–22. 10.1016/j.jvs.2018.10.052 30591293

[7] Arnaoutakis DJ Scali ST Beck AW Kubilis P Huber TS Martin AJ Comparative outcomes of open, hybrid, and fenestrated branched endovascular repair of extent II and III thoracoabdominal aortic aneurysms. J Vasc Surg. (2020) 71(5):1503–14. 10.1016/j.jvs.2019.08.236 31727462

[8] Schanzer A Simons JP Flahive J Durgin J Aiello FA Doucet D Outcomes of fenestrated and branched endovascular repair of complex abdominal and thoracoabdominal aortic aneurysms. J Vasc Surg. (2017) 66(3):687–94. 10.1016/j.jvs.2016.12.111 28259577

[9] Abdelhalim MA Tenorio ER Oderich GS Haulon S Warren G Adam D Multicenter trans-Atlantic experience with fenestrated-branched endovascular aortic repair of chronic post-dissection thoracoabdominal aortic aneurysms. J Vasc Surg. (2023) 78(4):854–62.e1. 10.1016/j.jvs.2023.05.053 37321524

[10] de Niet A Reijnen MM Tielliu IF Lardenoije JW Zeebregts CJ. Fenestrated endografts for complex abdominal aortic aneurysm repair. Surg Technol Int. (2016) 29:220–30. 27728949

[11] Tenorio ER Lima GB Marcondes GB Oderich GS. Sizing and planning fenestrated and branched stent-grafts in patients with chronic post-dissection thoracoabdominal aortic aneurysms. J Cardiovasc Surg. (2020) 61(4):416–26. 10.23736/S0021-9509.20.11365-X 32319275

[12] Scali ST Neal D Sollanek V Martin T Sablik J Huber TS Outcomes of surgeon-modified fenestrated-branched endograft repair for acute aortic pathology. J Vasc Surg. (2015) 62(5):1148–59.e2. 10.1016/j.jvs.2015.06.133 26254453 PMC5548461

[13] Gallitto E Faggioli G Pini R Logiacco A Mascoli C Fenelli C Reinterventions after fenestrated and branched endografting for degenerative aortic aneurysms. J Vasc Surg. (2021) 74(6):1808–16.e4. 10.1016/j.jvs.2021.05.027 34087395

[14] Dossabhoy SS Simons JP Diamond KR Flahive JM Aiello FA Arous EJ Reinterventions after fenestrated or branched endovascular aortic aneurysm repair. J Vasc Surg. (2018) 68(3):669–81. 10.1016/j.jvs.2017.12.036 29523438

[15] Doumenc B Mesnard T Patterson BO Azzaoui R De Préville A Haulon S Management of type IA endoleak after EVAR by explantation or custom made fenestrated endovascular aortic aneurysm repair. Eur J Vasc Endovasc Surg. (2021) 61(4):571–8. 10.1016/j.ejvs.2020.10.033 33414067

[16] Lounes Y Chassin-Trubert L Gandet T Ozdemir BA Peyron A Akodad M Endovascular aortic arch repair with a pre-cannulated double-fenestrated physician-modified stent graft: a benchtop experiment. Interact Cardiovasc Thorac Surg. (2021) 32(6):942–9. 10.1093/icvts/ivab023 34047348 PMC8932502

[17] Canaud L Ozdemir BA Chassin-Trubert L Sfeir J Alric P Gandet T. Double homemade fenestrated stent graft for total endovascular aortic arch repair. J Vasc Surg. (2019) 70(4):1031–8. 10.1016/j.jvs.2018.11.054 30922752

[18] Yang G Zhang M Zhang Y Du X Qiao T Li X Endovascular repair of postdissection aortic aneurysms using physician-modified endografts. Ann Thorac Surg. (2021) 112(4):1201–8. 10.1016/j.athoracsur.2020.11.016 33285129

[19] Tong YH Yu T Zhou MJ Liu C Zhou M Jiang Q Use of 3D printing to guide creation of fenestrations in physician-modified stent-grafts for treatment of thoracoabdominal aortic disease. J Endovasc Ther. (2020) 27(3):385–93. 10.1177/1526602820917960 32517556

[20] Tong Y Qin Y Yu T Zhou M Liu C Liu C Three-dimensional printing to guide the application of modified prefenestrated stent grafts to treat aortic arch disease. Ann Vasc Surg. (2020) 66:152–9. 10.1016/j.avsg.2019.12.030 31917223

[21] Major M Long GW Eden CL Studzinski DM Callahan RE Brown OW. Long-term outcomes and interventions of postoperative type 1a endoleak following elective endovascular aortic aneurysm repair. J Vasc Surg. (2022) 75(1):136–43.e1. 10.1016/j.jvs.2021.07.122 34324969

[22] Zuccon G D'Oria M Gonçalves FB Fernandez-Prendes C Mani K Caldeira D Incidence, risk factors, and prognostic impact of type Ib endoleak following endovascular repair for abdominal aortic aneurysm: scoping review. Eur J Vasc Endovasc Surg. (2023) 66(3):352–61. 10.1016/j.ejvs.2023.06.017 37356703

[23] Gennai S Andreoli F Leone N Bartolotti LAM Maleti G Silingardi R. Incidence, long term clinical outcomes, and risk factor analysis of type III endoleaks following endovascular repair of abdominal aortic aneurysm. Eur J Vasc Endovasc Surg. (2023) 66(1):38–48. 10.1016/j.ejvs.2023.03.018 36963748

[24] Marecki HL Finnesgard EJ Nuvvula S Nguyen TT Boitano LT Jones DW Characterization and management of type II and complex endoleaks after fenestrated/branched endovascular aneurysm repair. J Vasc Surg. (2023) 78(1):29–37. 10.1016/j.jvs.2023.02.016 36889609

[25] Canonge J Jayet J Heim F Chakfé N Coggia M Coscas R Comprehensive review of physician modified aortic stent grafts: technical and clinical outcomes. Eur J Vasc Endovasc Surg. (2021) 61(4):560–9. 10.1016/j.ejvs.2021.01.019 33589325

[26] Squizzato F Antonello M Modena M Forcella E Colacchio EC Grego F Fate of primary determinate and indeterminate target vessel endoleaks after fenestrated-branched endovascular aortic repair. J Vasc Surg. (2024) 79(2):207–16.e4. 10.1016/j.jvs.2023.09.036 37804955

[27] Kärkkäinen JM Tenorio ER Jain A Mendes BC Macedo TA Pather K Outcomes of target vessel endoleaks after fenestrated-branched endovascular aortic repair. J Vasc Surg. (2020) 72(2):445–55. 10.1016/j.jvs.2019.09.055 31980247

